# Assessment of Sedation in Mechanically Ventilated Children with Severe Acute Bronchiolitis: Correlation Between COMFORT-B Scale and Bispectral Index During Continuous Infusion of Fentanyl and Midazolam

**DOI:** 10.3390/medicina61111953

**Published:** 2025-10-30

**Authors:** Maj Jožef, Mojca Kerec Kos, Štefan Grosek, Melita Hajdinjak, Gregor Dolinar, Iztok Grabnar

**Affiliations:** 1 General Hospital Jesenice, Cesta Maršala Tita 112, 4270 Jesenice, Slovenia; majjozef@gmail.com; 2 Department of Biopharmaceutics and Pharmacokinetics, Faculty of Pharmacy, University of Ljubljana, Aškerčeva Cesta 7, 1000 Ljubljana, Slovenia; mojca.kerec-kos@ffa.uni-lj.si; 3 Department of Perinatology, University Medical Centre Ljubljana, Šlajmerjeva ulica 4, 1000 Ljubljana, Slovenia; stefan.grosek@kclj.si; 4 Department of Medical Ethics, Faculty of Medicine, University of Ljubljana, Vrazov trg 2, 1000 Ljubljana, Slovenia; 5 Laboratory of Applied Mathematics and Statistics, Faculty of Electrical Engineering, University of Ljubljana, Tržaška Cesta 25, 1000 Ljubljana, Slovenia; melita.hajdinjak@fe.uni-lj.si (M.H.); gregor.dolinar@fe.uni-lj.si (G.D.); 6 Institute of Mathematics, Physics and Mechanics, Jadranska Ulica 19, 1000 Ljubljana, Slovenia

**Keywords:** analgesia, sedation, fentanyl, midazolam, COMFORT-B, bispectral index, correlation

## Abstract

*Background and Objectives*: Analgesia and sedation are a major challenge in pediatric intensive care. The COMFORT-B scale and the Bispectral Index (BIS) are commonly used to assess the degree of sedation. The aim of this study was to investigate the correlation between the COMFORT-B and BIS and to evaluate the predictive validity of the BIS scale. *Materials and Methods*: Mechanically ventilated children (*n* = 41) diagnosed with acute bronchiolitis and treated with fentanyl and midazolam were included in the study. COMFORT-B and BIS scores were recorded over a 7-day observation period. Patients were divided into subgroups based on chronological age, neuromuscular blocker use, and level of sedation. Statistical analyses included correlation analysis by subject and time, simple moving average trend analysis, linear mixed-effects modeling and random forest. *Results*: Conventional correlation analysis revealed a weak to moderate correlation between the two scales in the entire cohort (Spearman rho of patients’ means 0.42, *p* = 0.007). The longitudinal correlation analysis by individual patient showed no significant relationship between the two scales in the entire cohort (CCF 0-lag 0.23; *p* = 0.33) or any subgroup. Linear mixed-effects model analysis showed that BIS score was associated with COMFORT-B score (slope = 0.799, *p* = 0.0002). The random forest model explained 19.6% of the variance. Both models yielded similar prediction errors (RMSE 10.6 and 11.3, respectively). *Conclusions*: We found a weak correlation between the two scales, which does not allow for reliable and valid predictions between the two scales. The BIS scale is suitable for the assessment of deep sedation, whereas the COMFORT-B scale is suitable for the assessment of moderate sedation.

## 1. Introduction

Sedation and analgesia are essential treatment modalities in the pediatric intensive care unit (PICU). Optimal sedo-analgesia has been shown to reduce pain and distress and is associated with better clinical outcomes and lower mortality [1,2]. Midazolam is a commonly used sedative agent in the PICU and is often used concomitantly with fentanyl due to its anxiolytic, amnestic, and synergistic sedative effects [3].

Sedation and analgesia in pediatric patients differ from those in adults as children cannot verbalize pain accurately [1]. Furthermore, sedation in children is essential to facilitate amnesia and movement control to ensure safe and successful completion of procedures such as magnetic resonance imaging or intubation [2]. Ideally, a sedated child is calm and asleep but can be easily awakened. Currently, there is no golden standard for the assessment of sedation in children. Consequently, analgesic and sedative medications are titrated empirically based on personal experience, physiological parameters and imprecise scales [2]. Conversely, excessive sedation is associated with an increased incidence of adverse events, including tolerance, respiratory depression and withdrawal symptoms, which may lead to a prolonged stay in the PICU. A review of the literature shows that adequate sedation is achieved in only 40% of critically ill children [4], with almost 50% of critically ill children experiencing pain which was not properly controlled in 15% of patients [5]. In addition, over-sedation occurs in 15% of children, particularly when neuromuscular blockers (NMB) are administered at the same time [6].

While clinical assessment remains essential, objective tools for recognizing different sedation levels are highly important. Sedation scales are a key clinical tool for assessing sedation in the PICU. The COMFORT-B scale and the Bispectral Index (BIS) are commonly used. The COMFORT-B scale is a modified version of the COMFORT scale that excludes physiological variables and focuses more on behavioral responses [7]. This modification is due to the lack of correlation between behavioral responses and clinical sedation outcomes when physiological variables are included [8]. It is the only validated scale for use in mechanically ventilated pediatric patients [9]. The scale consists of six behavioral categories (alertness, calmness/agitation, respiratory response, physical movement, muscle tone, facial muscles), each scored from 1 to 5 after a two-minute observation period. The COMFORT-B score is the sum of the individual category scores, with a range of 6 to 30 points. A score between 6 and 10 indicates over-sedation, 11–23 indicates moderate sedation, and 24–30 implies inadequate sedation [8]. The assessment is practical and patients do not need to be disturbed frequently. However, interrater bias is a major limitation. The COMFORT-B scale has not been validated in patients receiving continuous infusion of NMB [3].

The Bispectral Index (BIS) is an objective measure of brain activity derived from electroencephalography (EEG), with a range of 0–100. A value of 0 indicates no electrical activity in the brain, while a value of 100 indicates full awakening [10]. Lower values correspond to deeper levels of sedation, with a BIS value of ≤40 indicating deep sedation. Values between 40 and 60 are typically achieved during general anesthesia, while a range 64–80 is associated with an increased probability of amnesia [11]. The first validation was conducted in the context of intraoperative anesthesia in adults. The BIS scale has also been validated in pediatric patients requiring general anesthesia, with the exception of ketamine [12]. It is also widely used for the assessment of sedation in pediatric cardiac surgery, emergency diagnostic procedures, and even in pediatric dental patients [13,14,15]. The BIS score is significantly lower during sedation with propofol and in infants aged ≤6 months due to impaired synchronization of brain activity [16,17,18]. A moderate correlation of BIS with the University of Michigan Sedation Scale (UMSS) [12] and the Ramsay Sedation Scale (RSS) [19] has been demonstrated.

Literature data comparing the BIS and COMFORT-B scales are limited due to the introduction of less invasive and faster diagnostic procedures requiring shorter sedation duration and the use of agents such as dexmedetomidine [20] and ketamine [21] with alternative routes of administration [22,23]. Most reports consist of small cross-sectional and prospective cohort studies that have shown a moderate correlation between the two scales in a variety of surgical and diagnostic procedures, such as cardiac or gastrointestinal surgery, congenital heart disease, septic shock, and metabolic intoxication using a variety of anesthetics, even in older children and adolescents up to 18 years of age, which may shift BIS scores towards the adult population [9,24,25,26,27,28,29]. At least two studies have shown a strong correlation between the two scales in the range of deep to moderate sedation and concluded that the BIS scale is useful in the assessment of sedation to prevent oversedation [25,26]. On the other hand, other authors reported no significant correlation between the two scales [24].

The relationship between the BIS and COMFORT-B scales has therefore not yet been conclusively clarified. The aim of our work was to address this gap and determine whether a correlation exists between these two scales in mechanically ventilated children under two years of age treated with fentanyl and midazolam. Recognizing the limitations of previous cross-sectional studies, we conducted a longitudinal study in a homogeneous, age- and weight-restricted cohort with a consistent diagnosis of acute bronchiolitis over a seven-day observation window.

## 2. Materials and Methods

### 2.1. Study Design and Ethical Considerations

This prospective cohort study was part of a larger pharmacometric study, conducted between 2010 and 2013 in two tertiary university PICUs [30]. The study protocol was approved by the National Medical Ethics Committee of the Republic of Slovenia (ID 41/08/10). All procedures adhered to the ethical standards of the 1964 Declaration of Helsinki and its subsequent amendments. Written parental consent was obtained prior to the inclusion of each child in the study.

### 2.2. Study Population

The original cohort comprised 49 mechanically ventilated children of both sexes with a single diagnosis of acute bronchiolitis. They were treated with a prolonged intravenous infusion of fentanyl and midazolam over a period of at least three days. In addition, some patients received intermittent boluses of NMB. Children who weighed less than 3 kg, were older than 2 years or were treated with other opioids or ketamine were excluded. Children with neurological disease or brain injury were also excluded.

Assuming a moderate correlation between the BIS and COMFORT-B scores, statistical power analysis based on Fisher’s z-transformation indicated a minimum cohort size of 38 patients (R = 0.5, β = 0.2, α = 0.05).

### 2.3. Data Collection

The patients’ demographic and physiological clinical characteristics were taken from the medical charts and were described by chronological age, sex and body weight.

The primary clinical outcomes were COMFORT-B and BIS scores. The COMFORT-B score was assessed by PICU nurses who were blinded to the BIS results reported immediately afterwards. The COMFORT-B score was estimated at 15 min, 60 min and 12 h after the start of fentanyl and midazolam infusion and in the morning and afternoon (twice daily) on the remaining days of therapy within the seven-day observation window. The BIS value was determined before the start of the infusion and 5, 15, 60 min and 12 h after the start of the infusion initiation on the first day. Thereafter, it was measured twice daily, morning and afternoon, on the remaining days within the same observation window as the COMFORT-B score.

In addition, the concomitant pharmacotherapy was also documented with regard to possible drug interactions.

### 2.4. Statistical Analysis

Statistical analyses were performed using various methods, including correlation analysis by individual patient and time slices, simple moving average trend smoothing, linear mixed effects modeling and random forest.

All statistical analyses were performed using R software (version 4.2.0) and R Studio (version 2022.7.2.576, Posit Software, PBC) [31]. The cross-correlation analysis was performed using the rmcorr (version 0.7.0) [32] and TTR (version 0.24.3) [33] packages, while the packages lme4 (version 1.1-37) [34] and randomForest (4.7-1.2) [35] were used for regression analysis.

#### 2.4.1. Descriptive Statistics

A descriptive statistical analysis was performed for COMFORT-B scores and BIS. Counts and percentages were reported for categorical variables. Means and standard deviations were calculated for numeric variables. *p*-values for the difference between the means of group pairs were calculated using the non-parametric Wilcoxon rank sum test.

#### 2.4.2. Correlation Analysis

To assess the relationship between the BIS and COMFORT-B scores, the graphical representation of the time series was initially analyzed to assess a possible relationship between the two types of scores. As the BIS and COMFORT-B scores were available as a time series for each patient, we first calculated the means, time-weighted averages (TWA), medians, minima and maxima of both scores for each patient. Spearman correlation coefficients were calculated for time resolved BIS and COMFORT-B score for each patient and for each time point within the 7-day observation window. Children were further categorized into two subgroups, based on chronological age (<26 weeks and ≥26 weeks old), concomitant administration of NMB (with and without NMB) and COMFORT-B score (deep sedation 6–10 points and moderate sedation >10 points). The corresponding Spearman rho correlation coefficients were then calculated for the different subgroups.

To account for the timeliness of the data, cross-correlation function (CCF) analysis of time series was used to determine the relationship between the BIS and COMFORT-B scores for individual patient within the observation period in the uniform cohort and all subgroups [36]. The cross-correlation coefficient at lag 0 assesses the similarity of pairs of given time series, like the Pearson correlation coefficient, by pairing the data at identical times. Due to possible temporal inconsistencies in COMFORT-B and BIS score assessments each day, a correlation analysis for the entire cohort was performed on 15 time slices within an observation window of 7 days from the initiation of treatment. All cross-correlation coefficients with *p*-values of less than 0.05 were considered significant.

#### 2.4.3. Simple Moving Average Trend Analysis

Cox-Stuart trend analysis was used to detect the presence of a trend in order to minimize the effects of measurement uncertainty, testing the null hypothesis of no trend against the alternative hypothesis of a monotonic trend. The simple moving average (SMA) smoothing was used to estimate the underlying trend [37]. CCF of time series trends were then employed to improve the correlation between the COMFORT-B and BIS scores.

#### 2.4.4. Linear Mixed Effects

The predictive validity was assessed using linear mixed-effects modeling based on the maximum likelihood method, with the BIS score used as the dependent variable. The model included fixed and random effects on intercept and slope of the relationship between BIS and COMFORT-B score to estimate their typical population values and interindividual variability. Residual variability was modeled as additive, proportional and combination (additive + proportional) models. Covariate effects of patient’s age and sex, and NMB co-administration were tested as fixed effects on intercept and slope using likelihood ratio test (LRT) in a forward/backward stepwise method. Model performance (predictive criterion) was evaluated by root mean square error (RMSE) using leave-one-out cross-validation (LOOCV) [38].

#### 2.4.5. Random Forest

The flexible and robust random forest machine learning was also used to assess the non-linear predictive trends between the two scales [39]. The data were split by random sampling to obtain training (70%) and test (30%) data sets. Training sets were used for internal validation and test sets for external validation. Initial model training consisted of a hyperparameter grid. Hyperparameters tuning included the number of randomly sampled candidate variables (mtry), decision trees contained in the ensemble (ntree) and data points (nodesize) in a node required for the node to be split further. The model optimization consisted of internal 10-fold cross validation, repeated three times to improve accuracy and avoid overfitting. The black box approach features included age, sex, NMB co-administration and time. The importance of features on model prediction was based on the Gini index and Gini variable importance plots were developed for initial and reduced number of features [40]. Partial dependence plots (PDP) and individual conditional expectation (ICE) diagrams [41] were plotted for each feature to assess the impact of feature changes on dependent variable changes.

## 3. Results

**Figure 1:**
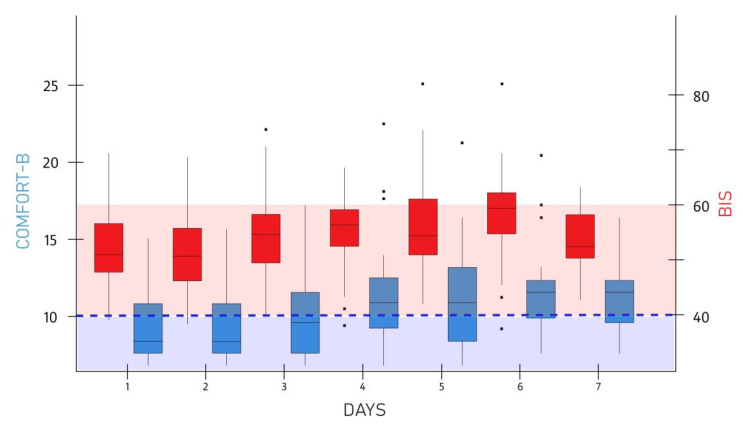
Box-plot series of daily BIS and COMFORT-B scores for the entire cohort of patients (*n* = 41). Red shaded area indicates moderate sedation by BIS, blue shaded area indicates deep sedation by COMFORT-B, broken blue line indicates COMFORT-B threshold for deep sedation.

The original cohort comprised 49 patients. Eight patients were excluded from further analysis due to inconsistent timing of the BIS and COMFORT-B scores estimation. The mean (SD) age and weight of the 41 patients (27 males/14 females) included in the analysis were 27.8 (37.3) weeks and 6.1 (2.9) kg, respectively. A box-plot series of BIS and COMFORT-B scores during the seven days of assessment is shown in Figure 1.

A total of 21 patients were found to be deeply sedated within a 7-day observation window (mean COMFORT-B ≤ 10), while according to the BIS score, only 1 patient was deeply sedated (mean BIS ≤ 40). A total of 35 children were moderately sedated (40 ≤ mean BIS ≤ 60). The mean COMFORT-B score of infants under 26 weeks of age was lower compared to older children (9.4 vs. 11.6; *p* = 0.017). Of the 21 deeply sedated children, 18 (86%) were younger than 26 weeks of age. A comparison of mean BIS revealed lower scores in infants aged 26 weeks or less (*p* = 0.053) and in the deeply sedated group based on the COMFORT-B score threshold (*p* = 0.026). The clinical characteristics of the entire cohort and subgroups are summarized in Table 1.

### 3.1. Statistical Analysis of the Entire Cohort

The results of the conventional correlation analysis showed a significant correlation between BIS and COMFORT-B with Spearman’s rho of the mean values and time-weighted averages (TWA) between 0.36 and 0.42. Cross-correlation function analysis demonstrated that 10 of the 41 cross-correlation function (CCF, zero lag) coefficients for the original, unsmoothed patient-specific time series were identified as significant. Of these, eight had positive values, ranging from 0.55 to 0.86, while the remaining two had negative values, with values of −0.66 and −0.69, respectively.

The CCF coefficients obtained after SMA smoothing demonstrated elevated correlations between the patient-specific time series with positive CCF coefficients reaching 0.97 and negative coefficients reaching −0.96. However, a significant correlation between the BIS and COMFORT-B scores time series was only observed in 12 of the 41 patients. Supplementary Table S1 summarizes an individual-based longitudinal correlation analysis. Results of the correlation analysis for 15 time slices of 12 h within a 7-day observation window are summarized in Table 2. The highest statistically significant Spearman correlation coefficient of 0.67 between the patients’ means was identified 7 days after the administration of fentanyl and midazolam (time slice 14).

### 3.2. Statistical Analysis of the Subgroups of Patients

Table 3 summarizes the correlation analysis in the whole cohort and subgroups, based on age, NMB administration and sedation level.

#### 3.2.1. Age

Spearman’s correlation showed a significant correlation (*p* < 0.05) between the two scales for mean scores in infants aged 26 weeks and younger and TWA (for both age subgroups), with Spearman’s correlation coefficients of 0.45 and 0.36, respectively (Table 3). CCF coefficients with zero lag showed a non-significant correlation between the two scales in both age subgroups (Table S1).

#### 3.2.2. Administration of Neuromuscular Blockers

A significant correlation between the two scales was found for the subgroup that did not receive intermittent boluses of NMB, with Spearman’s coefficients within a range of 0.36 to 0.61 for the means, medians, maxima and TWA (Table 3). The CCF coefficients with zero lag showed no significant correlation between the two scales in either NMB subgroup (Table S1).

#### 3.2.3. Degree of Sedation

Spearman’s coefficients in the deeply sedated subgroup (COMFORT-B ≤10) were significant in the range of 0.36 to 0.55 for the mean scores and TWA. No significant correlation was found in the moderately sedated group. A visual representation (Figure S1) of the time series of BIS and COMFORT-B scores (18 deeply sedated patients; 12 of whom were less than 26 weeks old, and 6 of whom were receiving intermittent boluses of NMB) illustrates that there is a considerable degree of diversity in the relationships between the time series pairs. No discernible patient-independent relationship was found between the BIS and COMFORT-B scores. In addition, the CCF coefficients with zero lag showed a non-significant correlation between the two scales in both subgroups.

### 3.3. Linear Mixed Effects

Linear mixed-effects modeling and random forest machine learning were used in the regression analysis, which showed that the BIS and COMFORT-B scores were significantly related (Table 4). A 1-point increase in COMFORT-B score was associated with a 0.799 increase in BIS. We were unable to estimate between-subject variability in the slope due to parameter shrinkage. Residual variability was most appropriately described by a proportional model. None of the covariates tested, including patient age and sex and concomitant administration of NMB had a significant effect. The residual error was 21.2%, indicating a lack of predictability between the BIS and COMFORT-B scores. RMSE of the final model was 10.6. Diagnostic plots (Figure S2) showed the adequate performance of the linear mixed-effects model.

### 3.4. Random Forest

The Grid search hyperparameter optimization included 1000 trees, 3 candidate variables and 5 data points in a node for evaluating the relationship between the BIS and COMFORT-B scores. The following features were included in the optimized model: age, time and COMFORT-B score. The optimized model explained only R^2^ = 19.6% of the variance, with a constant prediction error of 11.3 (Table S2). The impact of features on model prediction, based on Gini index are shown in Figure S3. PDP and ICE plots for the 3 features in the optimized model were created (Figure S4). Age pointed towards a higher degree of probability towards BIS score increase at an early age. COMFORT-B score in time showed a less pronounced inclination towards BIS score increase at higher COMFORT-B values at later stages of treatment.

## 4. Discussion

We conducted a prospective cohort study to investigate the correlation between the subjective COMFORT-B scale and the objective BIS scale in mechanically ventilated children with acute bronchiolitis who were sedated with fentanyl and midazolam. Sedation in the PICU is a challenge due to the heterogeneity and ethical concerns in this vulnerable patient group. Therefore, the level of sedation should be tailored to the individual needs of the patient.

The results of the BIS assessment showed that the majority of patients (85%) were categorized as moderately sedated, while the COMFORT-B scale showed that the majority of patients (51%) were categorized as deeply sedated. According to the BIS scale, only a few patients were categorized as over-sedated and none as under-sedated. One possible explanation for discrepancy between both scales is the subjective interpretation of the COMFORT-B score which is prone to significant interrater bias. In addition, the reliability of the clinical scales is affected by the influence of pharmacotherapy in the PICU (sedatives, neuromuscular blockers), which reduces muscle activity and response to stimuli. In our study, the COMFORT-B score was assessed by specialized PICU nurses. Previous literature has consistently shown a moderate to strong correlation in the assessment of sedation using clinical scales between physicians and specialized PICU nurses [22,27].

The COMFORT-B score was significantly lower in infants ≤26 weeks compared to older children. This may be attributed to the difficulty in accurately assessing the level of sedation in this age group. Conversely, BIS scores were not significantly lower in infants aged ≤26 weeks, which is inconsistent with the results of previous studies, suggesting that the lack of EEG synchronization in the former may contribute to this difference [18]. It is possible that the limited sample size of our cohort may have led to statistical non-significance.

The results of the conventional correlation analysis indicated a significant weak to moderate correlation using Spearman’s correlation coefficient of patient’s mean and time-weighted average between the COMFORT-B and BIS scales in the whole cohort and in the different subgroups, namely infants aged less than 26 weeks, children who did not receive intermittent NMB boluses and children who were deeply sedated. These results are comparable to those reported in the existing literature [9,25,26,27,28,29].

Spearman’s correlation coefficients do not consider the time series of the data and thus do not fully exploit the potential of our data set. An additional statistical analysis of the time series of BIS and COMFORT-B scores was then performed. The conclusions drawn from Spearman’s correlation coefficients for the means, TWA, medians, minima, and maxima of the patients’ BIS and COMFORT-B scores were confirmed and strengthened by more appropriate or powerful statistical methods, namely graphical representations, time series trend analysis, cross-correlation analysis, linear mixed-effects modeling and random forest. We believe that this comprehensive approach provides a high degree of accuracy and is compatible with the clinical context.

A longitudinal correlation analysis revealed a consistently weak overall correlation (Spearman) between the two scales, which is in contrast to the results of previous literature [9,25,26,27,28,29]. Our results are consistent with those of Amigoni et al. [24], who also reported a lack of correlation between the two scales. When we divided the patients into two age groups with a cut-off point of 26 weeks, no significant correlation (Spearman) was found between the two scales in either group. This is in contrast to the previous study, where a significantly higher correlation was found in patients aged ≤6 months compared to older children [20]. This finding may be due to the inherent difficulties in assessing sedation in infants aged ≤ 6 months.

Approximately half of the patients (54%) received intermittent boluses of NMB. Patients were therefore divided into two groups based on NMB administration. There were no significant differences between the two groups. This can be explained by the depletion of NMB prior to the COMFORT-B assessment.

Patients were then categorized into two different groups: deep and moderate sedation, based on the COMFORT-B 10-point threshold for deep sedation. In both groups, no significant correlation (Spearman) was found between the two scales. Therefore, it was not possible to distinguish between deep and moderate sedation using either scale as a reliable predictor. This is in contrast to the results of two other studies in which a significant correlation (r = 0.89) was found between the deep and moderate sedation groups [28,29]. It is hypothesized that the BIS scale is a surrogate marker for sedation and that it comprises only one dimension of the comfort paradigm, as the COMFORT-B scale assesses pain and distress. Second, this result may be attributed to the heterogeneity of the pediatric population and the lack of reference data. Third, the small number of patients in the deep sedation group may have contributed to the lack of statistical significance.

Reliable and valid predictions based on the two scales were only possible in about a quarter of our patients. Given the discrepancies in study results mentioned above, it is plausible that the discrepancies observed in previous studies are due to inherent limitations of these studies. These include the use of small sample sizes, the neglect of data dependence or cross-sectional correlation analysis between subgroups, and the failure to consider the timeliness of the data. Such approaches may lead to falsely increased correlation coefficients. In addition, other studies have been conducted on larger cohorts that included older children with a broader range of diagnoses, including surgical procedures, congenital heart disease, sepsis and toxicological exposures.

The BIS score is an unreliable method to differentiate between mild and moderate sedation. This is due to pronounced muscle artifacts that obscure the true BIS values and lead to falsely elevated values. This suggests that COMFORT-B may be a more accurate method of assessing moderate sedation in situations where clinical judgment is sufficient. Conversely, the BIS score appears to be a valuable tool in the assessment of deeply sedated children undergoing NMB infusion where responses to stimuli and physiological parameters are reduced. These patients need to be monitored electrophysiologically, as clinical assessment in deeply sedated and paralyzed children with altered physiological parameters is less reliable and associated with even higher interrater variability. This is supported by the findings of Aneja et al., who found that bedside assessment by nursing staff is inadequate in the area of deep sedation [19].

We decided to use linear mixed effects modeling because it enables us to assess interindividual and intraindividual variability in a sensitive population where data collection is sparse and timely inconsistent due to clinical interventions. The results of our study proved that both scales are significantly related although the predictive accuracy between both scales is sparse. Among covariates tested, we observed an insignificant trend of the decrease in the slope and an increase in the intercept of the BIS—COMFORT-B relationship with age. This is partly consistent with the literature reports, where the BIS score is significantly lower in infants less than 26 weeks of age [16,17,18]. The negative impact on the BIS temporal course can be interpreted by the sensitivity of immature developmental brain synchronization.

We have employed random forest machine learning since “black box” modeling approach is particularly suitable for limited data availability with high residual variability (i.e., statistical noise) and potential non-linear relationship between clinically important features. The results proved that age and time seem to be important clinical features; however, the model’s predictive accuracy remained low, indirectly favoring the linear mixed effects model due to its relative simplicity, confirming that simple models work better when performed within a highly variable, limited data collection. Utilizing random forest in our study has some merit. The relative insufficiency compared to the linear mixed effects model is suggestive that we may have not overlooked a significant correlation between the features and the dependent variable. This can only be confirmed through the analysis of larger data sets or, alternatively, by using carefully tuned linear models. The greatest strength of our study is its prospective design and the real-time insight it provides into clinical practice in the PICU. A single diagnosis of acute bronchiolitis confirms the uniformity of the cohort. Additionally, the observation window of seven days is longer than in other studies.

It should be noted that the present study has some limitations. First, the COMFORT-B scale was scored by multiple nurses, which may have introduced potential inter-rater bias. Second, the impedances of the BIS electrodes were not assessed, which may have significantly affected the quality of the data. Additional limitations of our study are the modest sample size and the lack of assessment of the impact of other concomitant medications on the COMFORT-B and BIS scores.

## 5. Conclusions

Sedation in the PICU remains a major challenge. Given the heterogeneity of the population, sedation in critically ill children is considered a crucial aspect that requires an individualized approach. The conclusion of our study in children on continuous infusion with fentanyl and midazolam is that the COMFORT-B and BIS scales are moderately associated in the deep sedation group, infants aged 26 weeks and younger and among children not receiving intermittent NMB boluses. None of the scales proved to be reliable in preventing over-sedation. The COMFORT-B and BIS scales assess different dimensions of discomfort as a clinical phenomenon. The clinical scores are interpreted subjectively with the COMFORT-B scale and are preferably used to differentiate between mild and moderate sedation. In contrast, the BIS can be used as a continuous and objective measurement for deeper levels of sedation with concomitant administration of NMB.

## Figures and Tables

**Table 1:** Clinical characteristics of all patients (*n* = 41) and subgroups, based on age, co-administration of neuromuscular blockers (NMB) and sedation level. Data are reported as mean (SD).

	All Patients	NMB Co-Administration			Age Group			Degree of Sedation		
	*n* = 41	Yes *n* = 22	No *n* = 19	*p*-Value	<26 Weeks *n* = 30	≥26 Weeks *n* = 11	*p*-Value	Deep Sedation COMFORT-B (6–10) *n* = 21	Moderate Sedation COMFORT-B > 10 *n* = 20	*p*-Value
Age (weeks)	27.8 (37.3)	35.5 (39.9)	18.9 (32.8)	0.014	8.1 (5.4)	81.6 (33.9)	<0.0001	28.9 (37.9)	31.7 (39.1)	0.025
Body weight (kg)	6.1 (2.9)	6.8 (3.0)	5.4 (2.6)	0.09	4.7 (1.2)	10.2 (2.2)	<0.0001	6.2 (2.9)	6.5 (2.9)	0.004
COMFORT-B	10.1 (2.7)	9.9 (2.3)	10.1 (3.1)	0.98	9.4 (2.6)	11.6 (2.5)	0.017	7.7 (1.1)	14.0 (2.3)	<0.001
BIS	51.5 (7.1)	51.0 (6.6)	51.9 (7.9)	0.77	50.1 (6.8)	54.8 (7.4)	0.053	50.7 (8.7)	55.5 (9.3)	0.026

**Table 2:** Time slice correlation analysis for the entire cohort (*n* = 41).

Time Slice *	*n*	Spearman Rho (*p*-Value)
1 2 3 4 5 6 7 8 9 10 11 12 13 14 15	37 37 39 37 35 34 35 35 30 25 23 22 14 10 9	0.41 (0.006) −0.11 (0.678) 0.18 (0.170) 0.10 (0.363) 0.17 (0.511) 0.37 (0.029) 0.39 (0.021) 0.06 (0.712) −0.10 (0.606) 0.07 (0.735) 0.48 (0.021) 0.33 (0.136) −0.09 (0.754) 0.67 (0.035) 0.21 (0.587)

* 12 h bins within a 7-day observation window.

**Table 3:** Spearman’s rank-order correlation of BIS with COMFORT-B score for the whole patient cohort and subgroups, based on age, NMB administration and sedation level. Data are presented as Spearman’s rank correlation coefficient (*p*-value).

Metric (by Subject)	All Patients	NMB Co-Administration		Age		Degree of Sedation	
	*n* = 41	Yes *n* = 22	No *n* = 19	<26 Weeks *n* = 30	≥26 Weeks *n* = 11	Deep COMFORT-B (6–10) *n* = 21	Moderate COMFORT-B > 10 *n* = 20
Mean	0.42 (0.007)	0.21 (0.34)	0.61 (0.0063)	0.45 (0.01)	0.29 (0.23)	0.55 (0.01)	0.083 (0.73)
Time weighted average	0.36 (0.022)	0.36 (0.022)	0.36 (0.022)	0.36 (0.022)	0.36 (0.022)	0.36 (0.022)	0.36 (0.022)
Median	0.31 (0.045)	0.12 (0.58)	0.47 (0.043)	0.33 (0.075)	0.27 (0.27)	0.37 (0.10)	0.13 (0.58)
Minimum	0.15 (0.34)	0.10 (0.66)	0.21 (0.40)	0.26 (0.16)	0.05 (0.85)	0.075 (0.75)	0.18 (0.45)
Maximum	0.40 (0.008)	0.29 (0.19)	0.51 (0.025)	0.35 (0.061)	0.21 (0.40)	0.34 (0.13)	0.24 (0.31)

**Table 4:** Parameter estimates of the linear mixed effects model of the relationship between the COMFORT-B and BIS as a dependent variable.

Parameter	Estimate	95% Confidence Interval
Intercept	43.1	(38.4, 47.9)
Slope	0.799	(0.383, 1.215)
**Between-subject variability (variance)**		
Intercept	25.8	(12.7, 38.8)
**Residual variability**		
Proportional error (%)	21.2	(19.1, 23.3)

## Data Availability

The original contributions presented in this study are included in the article/Supplementary Materials. Further inquiries can be directed to the corresponding author.

## Supplementary Materials

The following supporting information can be downloaded at: https://www.mdpi.com/article/10.3390/medicina61111953/s1, Table S1: Cross-correlation (CCF) results for the original time series of BIS and COMFORT-B scores and their simple moving average (SMA) trend components by individual patient; Table S2: Random forest hyperparameter optimization (ntree 1000, nodesize 5); Figure S1: Time series of BIS (red line) and COMFORT-B (blue line) scores for a group of deeply sedated patients <26 weeks of age (ID 25,28–31,32,34,35,38–41) and NMB co-administration (ID 28,29,34–37); Figure S2: Diagnostic plots of the linear mixed effects model. (A) Population predictions versus observations. (B) Individual predictions versus observations. (C) Predictions versus standardized residuals. (D) Q-Q plot of the standardized residuals; Figure S3: (A) Gini index for the model with 5 features. (B) Gini index for the optimized model with 3 features; Figure S4: Partial dependence plots (PDP) (thick line) and individual conditional expectation (ICE) plots (thin lines) for the three features in the optimized random forest.

## Abbreviations

The following abbreviations are used in this manuscript:

BIS	Bispectral Index
CCF	Cross-correlation function
COMFORT-B	A behavioral unobtrusive method of measuring distress in unconscious and ventilated infants, children and adolescence
EEG	Electroencephalography
ICE	Individual conditional expectation
LOOCV	Leave-one-out cross-validation
NMB	Neuromuscular blocker
PICU	Pediatric intensive care unit
RMSE	Root mean square error
SD	Standard deviation
