# Supplementary material for: Assessment of Sedation in Mechanically Ventilated Children with Severe Acute Bronchiolitis: Correlation Between COMFORT-B Scale and Bispectral Index During Continuous Infusion of Fentanyl and Midazolam
Source: Medicina (Kaunas). 2025 Oct 30;61(11):1953. doi: 10.3390/medicina61111953 (PMC12654302; doi:10.3390/medicina61111953)
Supplement: Supplementary file 1 [file medicina-61-01953-s001.zip › medicina-3904735-supplementary.pdf]

Assessment of Sedation in Mechanically Ventilated Children with Severe Acute Bronchiolitis: Correlation Between COMFORT-B Scale and Bispectral Index During Continuous Infusion of Fentanyl and Midazolam

Maj Jožef [1], Mojca Kerec Kos [2], Štefan Grosek [3,4], Melita Hajdinjak [5], Gregor Dolinar [5,6] and Iztok Grabnar [2,*]

1 General Hospital Jesenice, Cesta Maršala Tita 112, 4270 Jesenice, Slovenia; majjozef@gmail.com
2 Department of Biopharmaceutics and Pharmacokinetics, Faculty of Pharmacy, University of Ljubljana, Aškerčeva Cesta 7, 1000 Ljubljana, Slovenia; mojca.kerec-kos@ffa.uni-lj.si
3 Department of Perinatology, University Medical Centre Ljubljana, Šlajmerjeva ulica 4, 1000 Ljubljana, Slovenia; stefan.grosek@kclj.si
4 Department of Medical Ethics, Faculty of Medicine, University of Ljubljana, Vrazov trg 2, 1000 Ljubljana, Slovenia
5 Laboratory of Applied Mathematics and Statistics, Faculty of Electrical Engineering, University of Ljubljana, Tržaška Cesta 25, 1000 Ljubljana, Slovenia; melita.hajdinjak@fe.uni-lj.si (M.H.); gregor.dolinar@fe.uni-lj.si (G.D.)
6 Institute of Mathematics, Physics and Mechanics, Jadranska Ulica 19, 1000 Ljubljana, Slovenia
* Correspondence: iztok.grabnar@ffa.uni-lj.si; Tel.: +386-1-476-9543

Supplementary Materials

Table S1. Cross-correlation (CCF) results for the original time series of BIS and COMFORT-B scores and their simple moving average (SMA) trend components by individual patient. NMB - neuromuscular blocker.

ID	Age group < 26 weeks	NMB co-administration	COMFORT-B (6-10)	CCF zero lag coefficient (original)	*P*-value	CCF zero lag coefficient (SMA trends)	*P*-value
1	No	No	No	0.18	0.54	-0.19	0.56
2	Yes	Yes	Yes	0.33	0.29	0.35	0.33
3	No	Yes	No	-0.1	0.72	-0.46	0.11
4	No	Yes	No	0.34	0.26	0.46	0.15
5	Yes	Yes	Yes	0.53	0.053	0.70	0.011
6	Yes	Yes	Yes	0.61	0.11	-0.19	0.72
7	Yes	Yes	No	0.49	0.10	0.51	0.13
8	Yes	Yes	Yes	0.23	0.50	0.40	0.28
9	Yes	Yes	Yes	0.65	0.06	0.74	0.058
10	Yes	No	No	0.41	0.49	1.00	<0.0001
11	Yes	No	Yes	0.81	0.0025	0.34	0.37
12	No	Yes	No	-0.15	0.63	-0.34	0.31
13	No	No	No	-0.25	0.55	-0.39	0.45
14	No	Yes	Yes	0.16	0.71	-0.39	0.44
15	Yes	Yes	No	0.55	0.036	0.90	<0.0001
16	Yes	Yes	No	-0.69	0.04	-0.96	0.00052
17	Yes	Yes	No	-0.19	0.56	-0.32	0.37
18	Yes	No	No	0.48	0.14	0.79	0.011
19	Yes	No	Yes	0.18	0.57	-0.41	0.21
20	Yes	No	No	0.82	0.004	0.82	0.013
21	Yes	No	Yes	0.14	0.77	-0.73	0.16
22	Yes	Yes	Yes	0.81	0.0015	0.97	<0.0001
23	Yes	Yes	No	0.57	0.025	0.52	0.067

24	Yes	Yes	Yes	0.09	0.79	-0.04	0.91
25	Yes	No	Yes	-0.34	0.25	-0.28	0.41
26	No	Yes	No	0.33	0.47	0.81	0.10
27	No	Yes	Yes	0.24	0.53	0.50	0.25
28	Yes	Yes	No	0.25	0.59	-0.28	0.64
29	Yes	Yes	Yes	-0.44	0.17	-0.84	0.0046
30	Yes	No	Yes	-0.25	0.54	0.41	0.42
31	Yes	No	No	0.46	0.25	0.72	0.10
32	Yes	No	Yes	0.41	0.22	0.68	0.11
33	No	No	Yes	-0.43	0.15	-0.63	0.04
34	Yes	Yes	No	0.76	0.0015	0.96	<0.0001
35	Yes	Yes	Yes	0.86	0.012	0.93	0.023
36	No	Yes	No	0.11	0.73	-0.30	0.40
37	No	Yes	No	-0.03	0.94	-0.39	0.52
38	Yes	No	Yes	0.84	0.019	0.89	0.045
39	Yes	No	Yes	-0.66	0.0078	-0.80	0.001
40	Yes	No	Yes	0.42	0.26	0.71	0.076
41	Yes	No	No	-0.11	0.72	-0.37	0.24

Table S2. Random forest hyperparameter optimization (ntree 1000, nodesize 5). *MAE mean absolute error.

Hyperparameter	RMSE	R^2	MAE*
**ntree**			
1000	10.16	0.296	7.99
1500	10.18	0.301	8.15
2000	10.19	0.298	8.17
**mtry**			
1	11.32	0.196	9.06
2	11.51	0.198	8.92
3	11.63	0.195	8.97
4	11.75	0.187	9.01

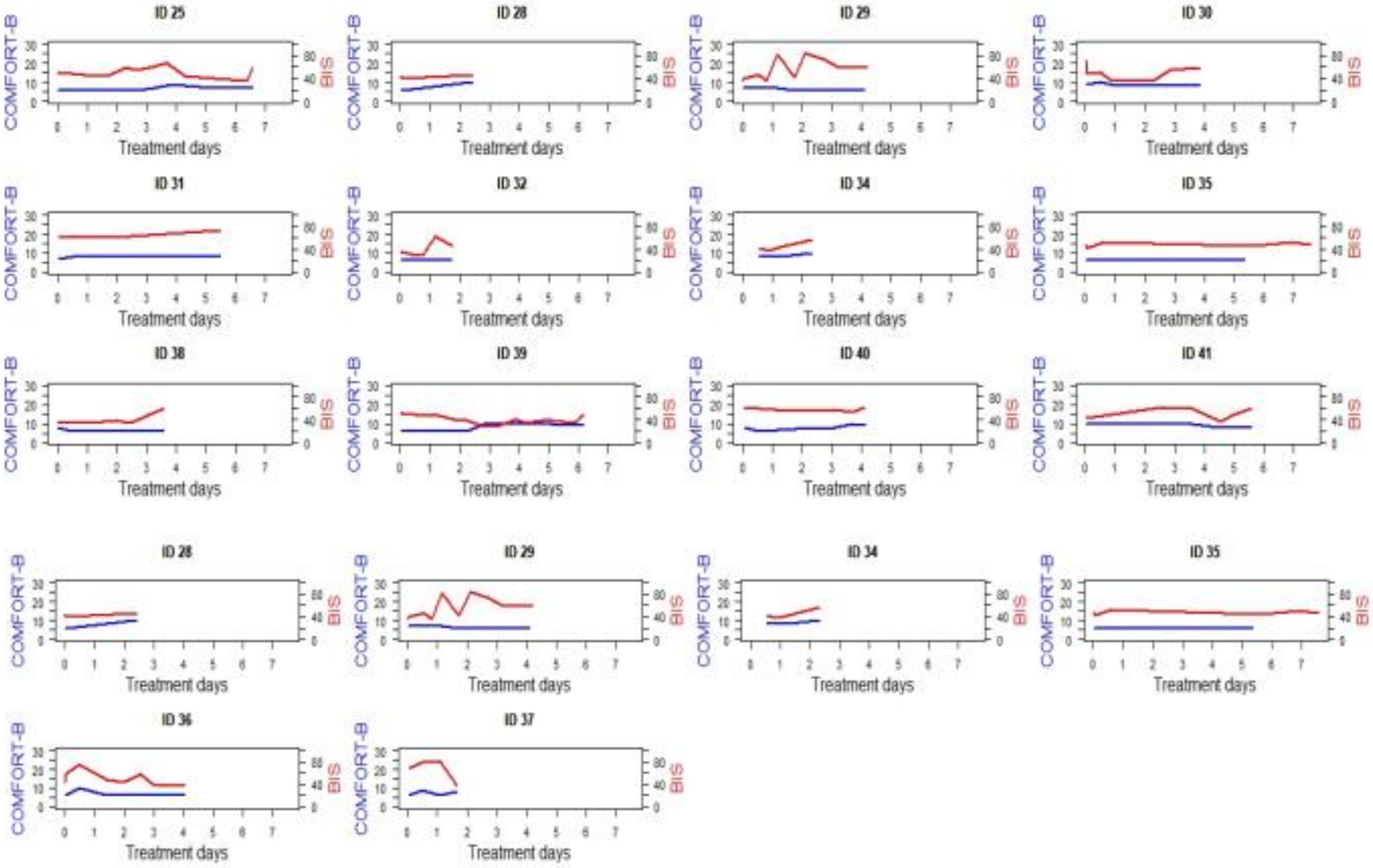

Figure S1. Time series of BIS (red line) and COMFORT-B (blue line) scores for a group of deeply sedated patients <26 weeks of age (ID 25,28-31,32,34,35,38-41) and NMB co-administration (ID 28,29,34-37).

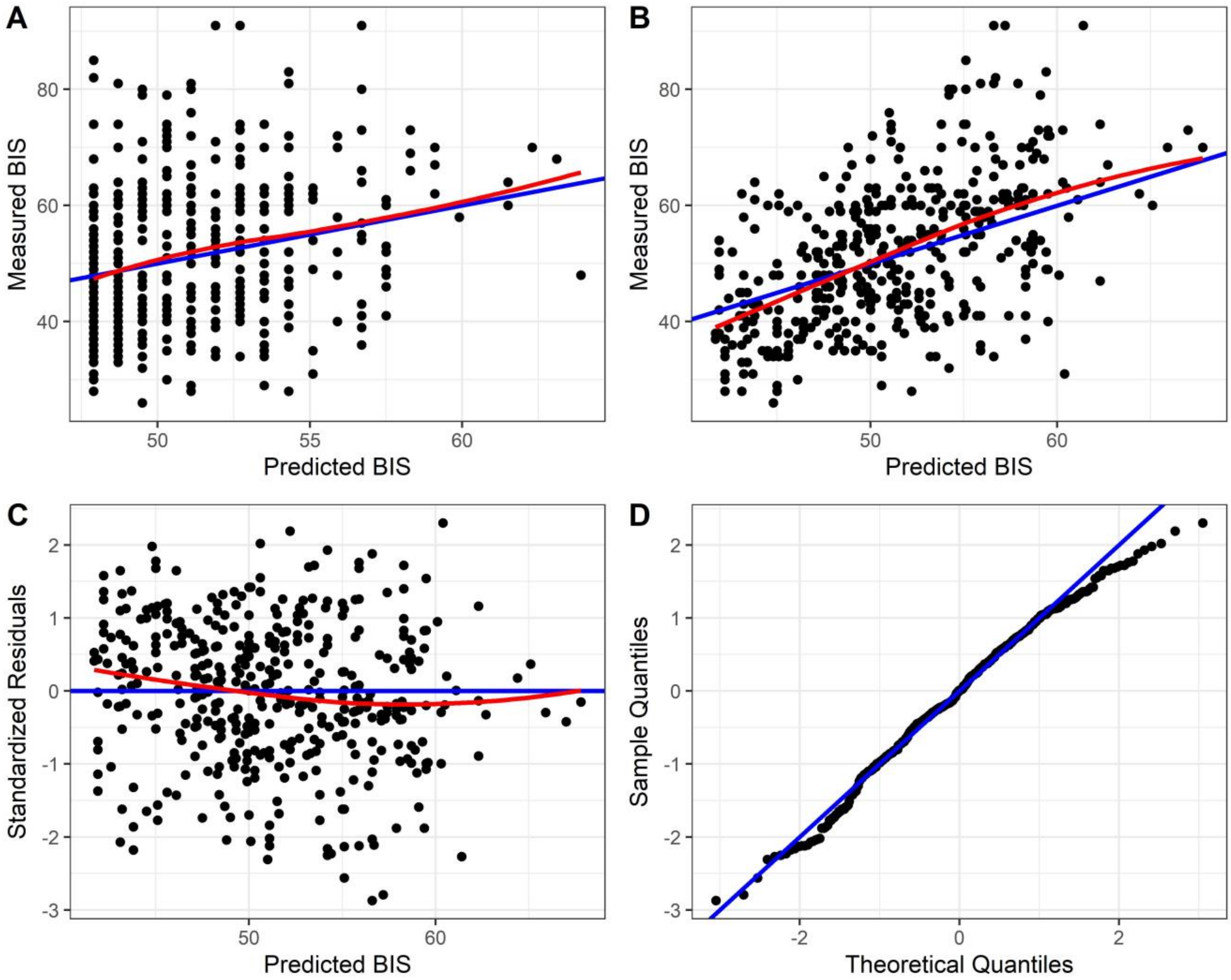

Figure S2. Diagnostic plots of the linear mixed effects model. **(A**) Population predictions versus observations. **(B**) Individual predictions versus observations. **(C**) Predictions versus standardized residuals. **(D**) Q-Q plot of the standardized residuals.

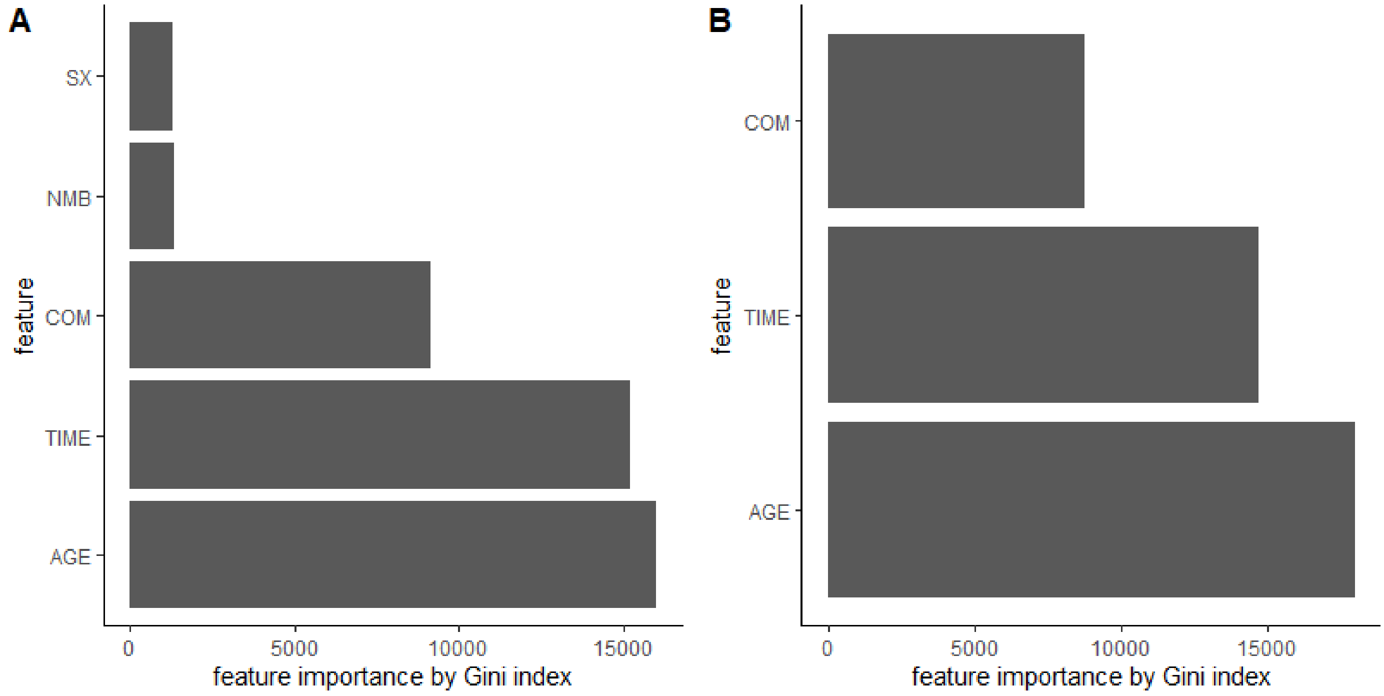

Figure S3. (**A**) Gini index for the model with 5 features. (**B**) Gini index for the optimized model with 3 features.

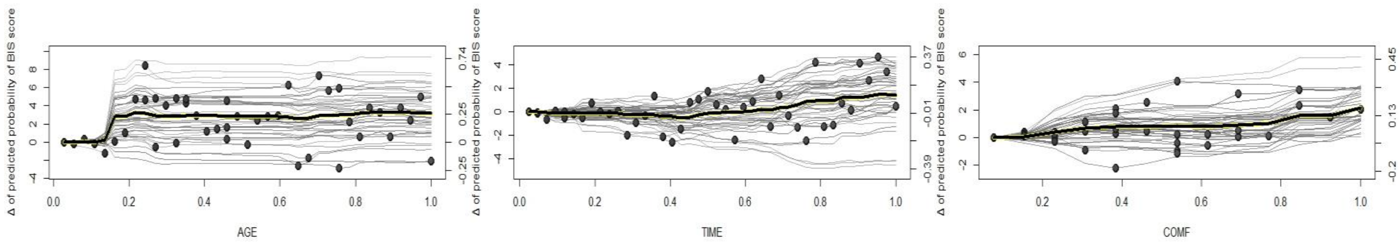

Figure S4. Partial dependence plots (PDP) (thick line) and individual conditional expectation (ICE) plots (thin lines) for the three features in the optimized random forest.
